# Serum and urine interferon-inducible protein 10, galectin-9, and SIGLEC-1 as biomarkers of disease activity in systemic lupus erythematosus

**DOI:** 10.55730/1300-0144.5804

**Published:** 2024-01-20

**Authors:** Şafak MİRİOĞLU, Suzan ÇINAR, Ömer ULUDAĞ, Erdem GÜREL, Sibel VARELCİ, Yasemin ÖZLÜK, Işın KILIÇASLAN, Yasemin YALÇINKAYA, Halil YAZICI, Ahmet GÜL, Murat İNANÇ, Bahar ARTIM ESEN

**Affiliations:** 1 Division of Rheumatology, İstanbul Faculty of Medicine, İstanbul University, Turkiye; 2 Graduate School of Health Sciences, İstanbul University, İstanbul, Turkiye; 3 Department of Immunology, Aziz Sancar Institute of Experimental Medicine, İstanbul University, İstanbul, Turkiye; 4 Division of Nephrology, School of Medicine, Bezmialem Vakıf University, İstanbul, Turkiye; 5 Department of Pathology, İstanbul Faculty of Medicine, İstanbul University, İstanbul, Turkiye; 6 Division of Nephrology, İstanbul Faculty of Medicine, İstanbul University, İstanbul, Turkiye

**Keywords:** Systemic lupus erythematosus, biomarker, IP-10, galectin-9, SIGLEC-1

## Abstract

**Background/aim:**

In this prospective study, we aimed to investigate the association of serum (s) and urine (u) IP-10, galectin-9, and SIGLEC-1 with disease activity in patients with systemic lupus erythematosus (SLE).

**Materials and methods:**

Sixty-three patients with active SLE (31 renal, 32 extrarenal) were included. Thirty patients with inactive SLE (15 renal, 15 extrarenal), 17 with renal active AAV, and 32 healthy volunteers were selected as control groups. Serum and urine IP-10, galectin-9, and SIGLEC-1 were tested using ELISA.

**Results:**

Levels of sIP-10 (p = 0.046), uIP-10 (p < 0.001), sGalectin-9 (p = 0.03), and uSIGLEC-1 (p = 0.006) were significantly higher in active SLE group compared to the inactive SLE; however, no differences were detected in the comparison of uGalectin-9 (p = 0.18) and sSIGLEC-1 (p = 0.69) between two groups. None of the biomarkers discriminated patients with active renal SLE from active extrarenal SLE. ROC analyses revealed an AUC of 0.63 (0.52–0.73) for sIP-10, 0.78 (0.68–0.86) for uIP-10, 0.64 (0.53–0.74) for sGalectin-9, and 0.68 (0.57–0.77) for uSIGLEC-1 in discriminating disease activity in SLE, which did not outperform C3 (0.75, 0.64–0.84) and C4 (0.72, 0.61–0.82). sIP-10 (p = 0.001), uIP-10 (p = 0.042), and uGalectin-9 (p = 0.009) were significantly increased in patients with active renal SLE compared to active renal AAV. sGalectin-9 (p < 0.001) and sIP-10 levels (p = 0.06) were decreased after 8 (5–22.5) months of treatment.

**Conclusion:**

sIP-10, uIP-10, sGalectin-9, and uSIGLEC-1 reflect global disease activity in SLE but do not outperform C3 and C4. sIP-10 and uIP-10 may be specific for active SLE compared to active AAV. sIP-10 and sGalectin-9 might be valuable in monitoring response after treatment.

## 1. Introduction

Systemic lupus erythematosus (SLE) is a multisystemic autoimmune disorder characterized by flares and remissions in its course [1]. Due to the unpredictable nature of the disease, patients continue to suffer from flares despite the significant improvement in patient care over the years [2,3]. Conventional biomarkers such as anti-dsDNA and serum levels of complement proteins, as well as indicators of specific active organ involvement like proteinuria and active urinary sediment for lupus nephritis (LN), have moderate sensitivity and specificity [2,3].

Interferons (IFNs) play a pivotal role in the pathogenesis of SLE. Autoantibodies and the nuclear antigens they are directed against form immune complexes that induce type 1 IFNs, released primarily by plasmacytoid dendritic cells [4]. Approximately 50% to 75% of patients with SLE are reported to have elevated gene signatures induced by IFN [5,6]. However, measurement of IFN signature is not standardized [4]. Using circulating IFN-α has not proved to be convenient as its serum levels were below the detection limit in several assays [4]. Not only IFN-α but also other type 1 IFNs, as well as type II and III IFNs, were demonstrated to contribute to the IFN signature [5,7]. Therefore, surrogate biomarkers reflecting IFN activity in the setting of active disease is needed, especially when new IFN targeting treatment options are considered.

Various studies investigated the association of IFN-inducible protein 10 (IP-10) with the IFN signature and disease activity in SLE [8–11], and a recent metaanalysis concluded that serum levels of IP-10 reflected systemic activity whereas urine levels could be useful for discriminating active LN [3]. However, most of these studies were cross-sectional and specificity of IP-10 for SLE was tested in just a few of them [3]. Galectin-9 was recently shown to be a promising biomarker in reflecting IFN and disease activity [4,5,12]. In the study by van den Hoogen et al., patients with an autoimmune disease other than SLE were involved as a control group [5]. Finally, evaluation of expression of sialic acid binding immunoglobulin-like lectin-1 (SIGLEC-1) on monocytes was reported to correlate with IFN; however, this procedure requires flow cytometry and intact cells and therefore is not feasible in clinical practice [13]. Only one study showed that serum levels of soluble SIGLEC-1 were correlated with its expression on monocytes but not with disease activity [13].

In this prospective study, we aimed to investigate the association of serum (s) and urine (u) levels of IP-10, galectin-9, and SIGLEC-1 with disease activity in patients with active SLE (renal and extrarenal) compared to patients with inactive SLE (renal and extrarenal), patients suffering from active renal disease of ANCA-associated vasculitis (AAV) and healthy volunteers.

## 2. Methods

### 2.1. Patient selection and data collection

All recruited patients with SLE fulfilled the Systemic Lupus International Collaborating Clinics (SLICC) and American College of Rheumatology (ACR) SLE classification criteria [14,15].

Disease activity was determined using SLE Disease Activity Index (SELENA-SLEDAI) based on the last 10 days of patients. Patients with a SLEDAI score of ≥4 or those exhibiting an organ/system-specific activity not covered by SLEDAI, such as autoimmune hemolytic anemia, were classified as ‘active’ [16,17]. When calculating SLEDAI, missing scores for anti-dsDNA antibody were treated as 0. Clinical SLEDAI (cSLEDAI) scores were also computed after immunological components (hypocomplementemia and positivity for anti-dsDNA antibodies) were omitted, and patients with a cSLEDAI score of ≥3 were classified as ‘clinically active’ [18]. Patients with active SLE who presented with at least one of four renal components of SLEDAI (renal SLEDAI, rSLEDAI)—proteinuria, hematuria, pyuria, urinary casts—were further classified as ‘active renal SLE’ (n = 31); otherwise, they were classified as ‘active extrarenal SLE’ (n = 32). Serum and urine specimens were re-collected from patients with active SLE after treatment.

**Figure 1:**
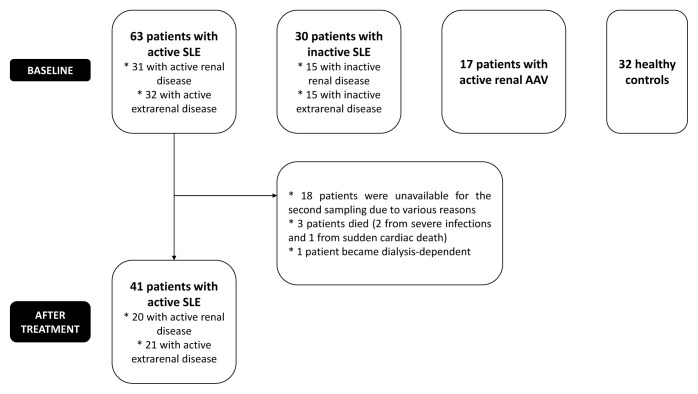
Flow chart of the study (AAV: ANCA-associated vasculitis, SLE: systemic lupus erythematosus).

The first control group consisted of patients with inactive SLE. Inactive disease was described as a SLEDAI score of <4 for the last ≥2 months. Patients with inactive SLE were further classified as ‘inactive renal SLE’ if they had biopsy-confirmed LN (n = 15), and as ‘inactive extrarenal SLE’ if they did not have LN (n = 15). The second control group included patients with AAV who had active renal disease (n = 17). All had biopsy-proven crescentic glomerulonephritis due to granulomatosis with polyangiitis (GPA) (n = 7) or microscopic polyangiitis (MPA) (n = 10) according to the 2012 International Chapel Hill Consensus Conference Nomenclature of Vasculitides [19]. Patients with AAV were recruited from our vasculitis outpatient clinic and cumulative data regarding disease characteristics were retrieved from vasculitis cohort database. At the time of sampling, clinical, laboratory, immunological, and therapeutic characteristics retrieved from the database were revised and disease activity was evaluated using Birmingham Vasculitis Activity Score (BVAS) version 3 [20]. All patients had active renal disease with a renal component score (renal BVAS) of ≥6. The healthy control (HC) group consisted of 32 volunteers. The flow chart of the study is illustrated in Figure 1.

The study was approved by the local ethical committee in İstanbul University (2019/547) and complied with the Declaration of Helsinki and its later amendments [21]. All participants provided written informed consent. Details on data collection; measurement of IP-10, galectin-9, and SIGLEC-1; and statistical analyses are provided in the supplementary materials.

## 3. Results

### 3.1. Baseline characteristics of patients

In total, 110 patients (93 SLE and 17 AAV) and 32 volunteers in the HC group were included. SLE and HC groups were comparable in terms of sex (84.9% and 71.9% female, respectively; p = 0.10) and age [35 (IQR: 27—47) and 31.5 (29–41) years, respectively; p = 0.66]. There were no significant differences between active renal and extrarenal SLE groups with regard to age [33 (26–47) and 33.5 (26–47) years, respectively; p = 0.80]. However, there were more female patients (96.9% and 74.2%, respectively; p = 0.01) and disease duration was slightly longer [76 (3–140.8) and 23 (0–90) months, respectively; p = 0.06) in the active extrarenal SLE group as compared to the active renal SLE group. At baseline, 76 patients with SLE (81.7%) were treated with immunosuppressive agents including corticosteroids, azathioprine, mycophenolate mofetil, rituximab, belimumab, methotrexate and calcineurin inhibitors. Fifty-three of 63 patients (84.1%) with active and 23 of 30 (76.7%) with inactive SLE were using immunosuppressives (p = 0.384). Baseline demographic, clinical and laboratory features of patients are shown in Table 1 and Table S1.

Arthritis (n = 15, 16.1%), rash (n = 13, 14%), immune cytopenias (n = 12, 12.9%), vasculitis (n = 10, 10.8%), and serositis (n = 7, 7.5%) were the most commonly affected domains of extrarenal activity. Immune cytopenias included 5 patients with thrombocytopenia and leukopenia, 4 with autoimmune hemolytic anemia, 2 with leukopenia, and 1 with thrombocytopenia only. Regarding serological activity, 48 patients (51.6%) had low complement levels and 39 (40.9%) had positive anti-dsDNA antibodies. Distribution of organ and system involvements according to SLICC criteria was comparable between active and inactive SLE. Median SLEDAI and cSLEDAI scores in the active SLE group were 10 (6–16) and 8 (4–12), while they were 0 (0–2) and 0 (0–0) in the inactive, respectively. Four patients were considered to be active based on non-SLEDAI items: three had autoimmune hemolytic anemia and one had severe neutropenia. In the active renal SLE group, median SLEDAI, cSLEDAI, and renal SLEDAI were 14 (10–16), 12 (8–12), and 8 (4–12), respectively.

Overall, 33 patients with SLE (35.5%) had damage. The median damage score of patients with damage was 1 (1–1). The most common domains of damage were avascular necrosis (n = 9, 9.7%), valvular heart disease (n = 8, 8.6%), pulmonary hypertension (n = 7, 7.5%), shrinking lung syndrome (n = 3, 3.2%), and cataracts (n = 3, 3.2%).

The active renal AAV group was different from the active SLE group in terms of sex distribution and age (p < 0.001 for both): 7 patients (41.2) were female and median age was 60 (48–65.5) years. Mean BVAS and renal BVAS were 16.5 ± 4.9 and 11.4 ± 1.5, respectively. Proteinuria and hematuria were present in 16 (94.1%) and 15 (88.2%) patients, respectively. Median serum creatinine and eGFR were 4.4 (1.6–6) mg/dL and 13.9 (7.6–46.1) mL/min/1.73 m^2^, respectively (p < 0.001 for both when compared to the active SLE group). Comparison of proteinuria [2.1 (1.3–4.8) vs 2.2 (1.4–3.9) g/g; p = 0.96] and serum albumin levels (3 ± 0.79 vs 3.4 ± 0.57 g/dL; p = 0.15) between patients with active renal SLE and AAV did not reveal any significant differences. General symptoms (n = 12, 70.1%) such as myalgia, arthritis/arthralgia, fever and weight loss, and pulmonary involvement (n = 8, 47.1%) were the most common domains of extrarenal activity in patients with AAV.

### 3.2. Serum and urine levels of IP-10, galectin-9, and SIGLEC-1

**Figure 2:**
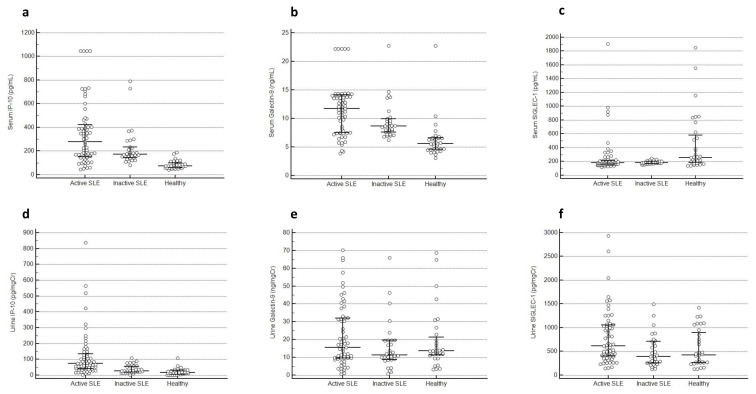
Serum **(a–c)** and urine **(d–f)** levels of all biomarkers in active SLE, inactive SLE, and healthy control groups. Detailed statistics are provided in the text and tables (IP-10: interferon-inducible protein 10, SIGLEC-1: sialic acid binding immunoglobulin-like lectin-1, SLE: systemic lupus erythematosus).

Serum and urine levels of IP-10, galectin-9, and SIGLEC-1 across various study groups are detailed in Tables 2, S2, and S3. sIP-10 levels were higher in the active SLE group [279.4 (147.5–430.4) pg/mL] compared to the inactive SLE group [173.4 (142.3–247.9) pg/mL, p = 0.046] and HC group [74.4 (58.8–103) pg/mL, p < 0.001] (Figure 2a). Analyses of subgroups demonstrated that sIP-10 levels were similar between patients with the active renal and the extrarenal SLE groups (p = 0.24), and the active renal and the inactive renal SLE groups (p = 0.36). sGalectin-9 was found to be higher in patients with active SLE [11.7 (7.5–14.1) ng/mL] when compared to inactive SLE [8.7 (7.5–10) ng/mL, p = 0.03] and HC groups [5.6 (4.6–6.6) ng/mL, p < 0.001] (Figure 2b). sGalectin-9 levels were also similar between patients with active renal and extrarenal SLE (p = 0.49). Despite higher absorbance values of sGalectin-9 in active renal SLE compared to inactive renal SLE, the difference was not statistically significant (p = 0.09). sSIGLEC-1 levels were not higher in the active SLE group [181.2 (157.9–213.9) pg/mL] when compared with the inactive SLE group [182.6 (169.9–203.1) pg/mL, p = 0.69] and lower compared to the HC group [258.5 (179–602.1) pg/mL, p = 0.001] (Figure 2c). sSIGLEC-1 levels did not differ between patients with active renal and extrarenal SLE (p = 0.91), and active renal and inactive renal SLE (p = 0.39). When compared with active renal AAV, sIP-10 levels were significantly higher in patients with active renal SLE (p = 0.001) (Figure S1a). However, sGalectin-9 and sSIGLEC-1 had similar levels (p = 0.64 and p = 0.07, respectively) (Figures S1b and S1c).

uIP-10 levels were higher in the active SLE group [73.5 (40.9–136.9) pg/mgCr] compared to the inactive SLE [26.1 (18.1–55.2) pg/mgCr, p < 0.001] and the HC groups [16.5 (5.1–32.5) pg/mgCr, p < 0.001] (Figure 2d). uIP-10 levels were similar between patients with active renal and extrarenal SLE (p = 0.32). Patients with active renal SLE had higher levels of uIP-10 when compared to those with inactive renal SLE [83.2 (33.1–180.3) pg/mgCr and 26.3 (20.9–58.4) pg/mgCr, p = 0.006]. uGalectin-9 levels were not higher in patients with active SLE [15.5 (9.6–32.1) ng/mgCr] as compared to the inactive SLE [11.4 (8.8–19.5) ng/mgCr, p = 0.18] and the HC groups [13.6 (11.3–22.1) ng/mgCr, p = 0.76] (Figure 2e). Additionally, uGalectin-9 levels were similar between patients with active renal and inactive renal SLE. However, patients with active extrarenal SLE had higher uGalectin-9 levels than those with active renal SLE (p = 0.02). uSIGLEC-1 levels were increased in the active SLE group [619.7 (389.4–1056.6) pg/mgCr] compared with the inactive SLE [393.2 (248.6–715.8) pg/mgCr, p = 0.006] and the HC groups[425.7 (264.7–925.9) pg/mgCr, p = 0.04] (Figure 2f). Further analyses showed that uSIGLEC-1 levels were similar between the active renal and the extrarenal SLE groups (p = 0.45). Patients with active renal SLE had higher uSIGLEC-1 levels than those with inactive renal SLE (p = 0.05). When compared with the active renal AAV group, the active renal SLE group exhibited higher levels of uIP-10 (p = 0.04) and uGalectin-9 (p = 0.009), along with lower levels of uSIGLEC-1 (p = 0.004) (Figures S1d–S1f).

Serum C3 and C4 levels were lower in the active SLE group compared to the inactive SLE group (p < 0.001 and p = 0.002, respectively). Additionally, these levels tended to discriminate active renal from active extrarenal disease. Anti-dsDNA positivity was similar between the active and the inactive SLE groups (p = 0.88), but results were available for only 4 patients with inactive SLE (Table S2).

Levels of all biomarkers were similar in patients with SLE with regard to the use of immunosuppressive agents at baseline (Table S4).

### 3.3. Correlation of biomarkers with various features

Serum IP-10 (r = 0.19, p = 0.07) and serum galectin-9 (r = 0.17, p = 0.11) were very weakly, urine SIGLEC-1 (r = 0.27, p = 0.009) was weakly, and urine IP-10 (r = 0.48, p < 0.001) was moderately correlated with SLEDAI. Coefficients with cSLEDAI were higher in sIP-10, uIP-10, and sGalectin-9, and slightly lower in uSIGLEC-1 preserving the significance. Correlation with SLEDAI resulted in higher coefficient values compared to renal SLEDAI in all biomarkers (Table S5).

uIP-10 had a negative correlation with age (r = −0.23, p = 0.03), serum albumin (r = −0.46, p = 0.006), serum C3 (r = −0.46, p < 0.001), and serum C4 (r = −0.38, p < 0.001). Moreover, uIP-10 showed correlation with proteinuria (r = 0.37, p = 0.01).

### 3.4. Performance of biomarkers in predicting disease activity

Receiver operating characteristic (ROC) analyses revealed good AUC values for sIP-10 [area under the curve (AUC): 0.63, 95% confidence intervals (CI): 0.52–0.73, p = 0.03] and sGalectin-9 (AUC: 0.64, 95% CI: 0.53–0.74, p = 0.02) in discriminating disease activity in patients with SLE (Figure S2a). Optimal threshold values were calculated as 299.5 pg/mL for sIP-10 (49.2% sensitivity and 86.7% specificity) and 10.2 ng/mL for sGalectin-9 (61.9% sensitivity and 80% specificity).

uIP-10 and uSIGLEC-1 performed well in discriminating disease activity in SLE, as well. uIP-10 had an AUC of 0.78 (95% CI: 0.68–0.86, p < 0.001) and uSIGLEC-1 had an AUC of 0.68 (95% CI: 0.57–0.77, p = 0.003) (Figure S2b). Optimal threshold values were determined as 36.9 pg/mgCr for uIP-10 (79.4% sensitivity and 70% specificity) and 417.7 pg/mgCr for uSIGLEC-1 (74.6% sensitivity and 56.7% specificity).

On the other hand, serum C3 had an AUC of 0.75 (95% CI: 0.64–0.84, p < 0.001) and serum C4 had an AUC of 0.72 (95% CI: 0.61–0.82, p < 0.001). Optimal threshold values were 61 mg/dL for serum C3 (44.1% sensitivity and 100% specificity) and 10 mg/dL for serum C4 (58.33% sensitivity and 82.61% specificity). Positivity for anti-dsDNA antibodies had an AUC of 0.52 (95% CI: 0.38–0.66, p = 0.89) with a 28.6% sensitivity and 75% specificity; however, the test was not performed in 43% of patients at the time of sampling (Figures S3a and S3b).

Further ROC analyses revealed that no candidate biomarkers were successful in distinguishing patients with active renal from active extrarenal disease in patients with SLE (data not shown).

According to cSLEDAI, 50 of 63 patients (79.4%) with active SLE were classified as ‘clinically active’. ROC analyses still showed good AUC values for sIP-10 (AUC: 0.62, 95% CI: 0.51–0.72, p = 0.04), but not for sGalectin-9 (AUC: 0.60, 95% CI: 0.49–0.70, p = 0.09) in discriminating clinical disease activity in patients with SLE. Optimal threshold value was calculated as 347.9 pg/mL for sIP-10 (45.3% sensitivity and 85% specificity). uIP-10 performed well in discriminating clinical disease activity in SLE, while uSIGLEC-1 did not. uIP-10 had an AUC of 0.77 (95% CI: 0.67–0.85, p < 0.001) and uSIGLEC-1 had an AUC of 0.60 (95% CI: 0.49–0.70, p = 0.09). Optimal threshold value was determined as 36.9 pg/mgCr for uIP-10 (81.1% sensitivity and 60% specificity).

### 3.5. Biomarker levels after treatment

**Figure 3:**
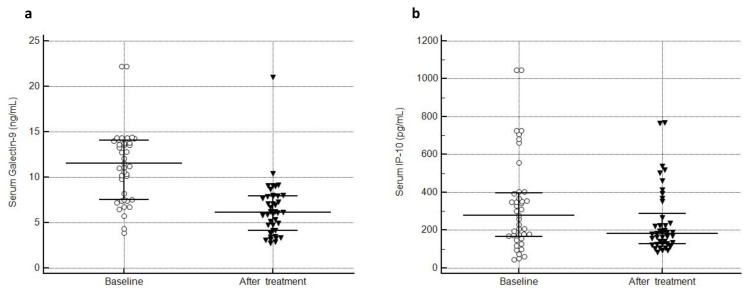
Serum galectin-9 **(a)** and IP-10 **(b)** at baseline and after treatment in patients with active SLE (IP-10: interferon-inducible protein 10, SLE: systemic lupus erythematosus).

Of 63 patients with active SLE, 41 patients were re-tested for serum and urine levels of all biomarkers after a median treatment duration of 8 (5–22.5) months. Eighteen patients were unavailable for the second sampling, 3 died (2 from severe infections and 1 from sudden cardiac death), and 1 became dialysis-dependent. Median SLEDAI and cSLEDAI decreased from 10 (6–15.5) and 8 (4–12) to 2 (0–4) and 0 (0–4), respectively, after treatment (p < 0.001 for both). sGalectin-9 levels were significantly lower after treatment [6.1 (4.1–7.9) ng/mL] as compared to baseline [11.5 (7.5–14.1), p < 0.001] (Figure 3a). sIP-10 levels also decreased, yet this difference did not reach statistical significance (p = 0.06) (Figure 3b). C3 (p = 0.04) and C4 (p = 0.004) increased after treatment, and anti-dsDNA positivity (p = 0.002) declined. Serum and urine levels of all biomarkers in 41 patients with active SLE are shown in Table S6.

## 4. Discussion

Pathogenesis of SLE involves a disruption in immune tolerance culminating in autoreactive lymphocytes and activation of the innate immune system including the complement cascade [22]. Immune complexes formed by autoantibodies and their target antigens activate type 1 IFN system which is considered to play a major role in disease pathogenesis [4]. Due to the limited success of conventional biomarkers, such as levels of serum complement proteins and anti-dsDNA antibodies, as well as parameters like serum creatinine, proteinuria, and urinary sediment for renal lupus, which are used in daily practice to indicate systemic disease activity or organ/system involvement, there has been a push to search for new surrogate biomarkers of disease activity [23]. In recent years, this search has started to focus on several cytokines and chemokines reflecting both IFN and disease activity given the new treatment options being currently investigated in trials [24]. Among several molecules, IP-10, galectin-9, and SIGLEC-1 differentiate themselves as potential biomarkers of IFN and disease activity in SLE and LN [3,5,13]. In this study, we demonstrated that serum and urine IP-10, sGalectin-9, and uSIGLEC-1 discriminated patients with active SLE. Moreover, serum and urine IP-10 displayed specificity for active renal SLE as compared to active renal AAV. Nevertheless, they did not outperform serum C3 and C4. None of the biomarkers were able to distinguish active renal from active extrarenal SLE.

IP-10 and its receptor CXCR3 are primarily expressed by activated T helper cells, B cells, and macrophages and secreted in response to IFN stimulation [3,25]. Binding of IP-10 to CXCR3 is responsible for lymphocyte trafficking to the affected organs and tissues in SLE which was shown in murine models [3]. Although serum IP-10 has been shown to be a promising biomarker in several studies [8,25], there are also conflicting results reported in different cohorts [4]. A recent metaanalysis demonstrated that sIP-10 reflected systemic activity and uIP-10 was useful for detecting active LN [3]. In this study, we found that sIP-10 and uIP-10 performed well in discriminating overall disease activity in SLE. Moreover, sIP-10 was decreased after successful treatment.

Galectin-9 is a ubiquitously expressed and produced beta-galactoside binding lectin with multiple immunomodulatory functions including maturation of dendritic cells (DCs), expansion of regulatory T cells, apoptosis of activated T cells and modulation of Th1 and Th17 immunity [26–29]. Galectin-9 expression was found be increased in DCs of patients with SLE and high IFN activity [5,30,31]. Furthermore, knockout of LGALS9 ameliorated nephritis and arthritis in a murine model, which all suggested that galectin-9 may have a role in disease pathogenesis [5,30,31]. van den Hoogen et al. demonstrated that sGalectin-9 was a good biomarker for active LN in 2018 [5], and this finding was supported by different cohorts [12,27]. Results of the only study that investigated uGalectin-9 as a biomarker were not promising [27]. Our findings of serum and urine galectin-9 were in line with previous studies. Moreover, we showed that levels of serum sGalectin-9 significantly diminished after treatment.

SIGLEC-1 is a cell adhesion molecule which is mainly expressed in tissue-resident macrophages and monocyte-derived dendritic cells [32,33]. Its expression is profound in circulating CD14^+^ monocytes which was reported to be increased in various autoimmune conditions [34,35]. Several studies demonstrated that expression of SIGLEC-1 as a surface molecule of monocytes was a promising biomarker [9,10,36,37]; however, only Oliveira et al. investigated serum levels of soluble SIGLEC-1 in SLE and concluded that sSIGLEC-1 reflected the expression on monocytes that was not found to be correlated with disease activity [13]. To the best of our knowledge, uSIGLEC-1 has not been evaluated before. In our study, sSIGLEC-1 was not able to show disease activity in SLE; however, uSIGLEC-1 discriminated active SLE from inactive SLE. Nevertheless, urine levels did not distinguish active renal from active extrarenal SLE and no change was observed after treatment.

ROC analyses demonstrated that even though sIP-10, uIP-10, sGalectin-9, and uSIGLEC-1 were successful at discriminating disease activity in SLE. They also showed that serum C3 and C4 levels performed better with higher AUC levels except for uIP-10. When the active disease state was determined by using only cSLEDAI, sGalectin-9, and uSIGLEC-1 were found to not perform well although coefficients showed a correlation. However, it should be kept in mind that cSLEDAI comes with the innate limitations originating from SLEDAI, such as failure to reflect important clinical activity domains like autoimmune hemolytic anemia which was present in our patients with active SLE [38]. None of the biomarkers tested in this study were able to discriminate active renal from active extrarenal SLE. Correlation analyses revealed higher coefficient values with SLEDAI compared to renal SLEDAI, which suggested that sIP-10, uIP-10, sGalectin-9, and uSIGLEC-1 may have value in reflecting global disease activity rather than organ or system specific activity.

Inclusion of patients with active renal involvement of AAV enabled us to test the specificity of these biomarkers. To the best of our knowledge, IP-10 was tested in comparison to other autoimmune diseases in a few studies [3], galectin-9 was only compared to patients with primary APS [5], and soluble SIGLEC-1 was evaluated against systemic sclerosis [13]. Despite the very different pathogenetic backgrounds of SLE and AAV, we think that a systemic disease affecting the kidneys may be a good comparison for SLE considering the magnitude of inflammation emerging with the disease. Supporting this assumption, patients in the active renal AAV group had high activity scores according to those in the BVAS group. Serum and urine IP-10 levels were significantly higher in the active renal SLE group as compared to the active renal AAV group. Moreover, although uGalectin-9 was not successful in discriminating disease activity in the SLE group, its levels were profoundly lower in the active renal AAV group. In summary, serum and urine IP-10 and uGalectin-9 may have specificity for SLE. However, we are also aware that patients in the AAV group had deteriorated kidney functions, which may have affected these findings, but we also consider the fact that AAV commonly present with acute kidney injury [39].

Correlation studies revealed that uIP-10 showed a positive correlation with proteinuria. Beyond any reflection of the active disease state, free excretion associated with proteinuria can be another explanation for the high biomarker levels in the urine. However, higher correlation coefficient values with serum C3 and C4 compared to proteinuria may suggest that the latter possibility is unlikely.

This study has some limitations. First, it would have been better to increase the number of patients. Second, our study lacks results regarding IFN gene signatures. Thus, it is not possible to know whether our findings with biomarkers fully reflect IFN activity. Third, we did not evaluate the expression of SIGLEC-1 on monocytes; therefore, it may not be certain that our results with SIGLEC-1 are correlated with its expression on cell surface. Fourth, the elder median age of patients with AAV in our study may have an effect on biomarker levels in those patients. Moreover, comparing two groups with considerable differences in renal functions may be a concern. Finally, results of anti-dsDNA were not available in 43% of patients with SLE, most of whom belonged to the inactive group. However, cSLEDAI scores in patients with inactive SLE were already 0, and comparison with the active group was very significant.

On the other hand, our study has several strengths. First, it was designed as a longitudinal study. Biomarkers were re-tested after treatment reaching lower disease activity in most cases. Second, we had well-chosen control groups to evaluate not only the sensitivity to detect active disease but also the specificity of the biomarkers. While the number of patients could have been higher, participants were recruited from dedicated SLE/APS and vasculitis outpatient clinics with an established cohort of patients who were regularly monitored by experienced clinicians. These clinicians also contributed data for international collaborations. Finally, to the best of our knowledge, this is the first study evaluating urine soluble SIGLEC-1 as a biomarker of disease activity in SLE.

In conclusion, serum and urine IP-10, sGalectin-9, and uSIGLEC-1 seem to be promising biomarkers reflecting global disease activity in patients with SLE although they do not outperform serum C3 and C4. Serum and urine IP-10 may be specific for active SLE as compared to active AAV. sIP-10 and sGalectin-9 might be valuable in monitoring response after treatment for active disease. Larger multicenter studies with appropriate control groups are needed to further evaluate these biomarkers.

## Supplementary Methods

### Data Collection

Patients registered after 1991 were followed up using a standard protocol in the weekly SLE outpatient clinic by the same clinicians. This protocol consisted of data on demographic characteristics, SLE classification criteria, mortality, autoantibody profile, treatment history, antiphospholipid syndrome classification criteria, features of nephritis including histopathology, and Systemic Lupus International Collaborating Clinics / American College of Rheumatology (SLICC/ACR) damage index [1]. For the purpose of this study, demographic characteristics, cumulative clinical and laboratory features, autoantibody profiles, renal histopathology, damage data and treatment history were retrieved from the database and revised at the time of first sampling. Duration of disease was defined as the time from the diagnosis of SLE to the time of first sampling and duration of follow-up was defined as the time from the first visit to the time of first sampling in our SLE outpatient clinic.

Patients with ANCA-associated vasculitis were recruited from our vasculitis outpatient clinic and cumulative data regarding disease characteristics were retrieved from vasculitis cohort database. At the time of sampling, clinical, laboratory, immunological and therapeutic characteristics retrieved from the database were revised.

In patients with renal involvement, proteinuria was determined using urinary protein-to-creatinine ratio (g/g) in the first morning specimens. Estimated glomerular filtration rate (eGFR) was calculated with Chronic Kidney Disease Epidemiology Collaboration (CKD-EPI) 2009 formula [2]. Kidney biopsies of patients with SLE were reviewed according to International Society of Nephrology / Renal Pathology Society (ISN/RPS) 2003 classification system by nephropathologists [3].

### Measurement of IP-10, Galectin-9 and SIGLEC-1

Serum and first morning urine specimens were collected from each patient and centrifuged at 3000 rpm for 10 minutes and 3500 g for 3 minutes, respectively. Aliquots prepared were stored at −80 °C until further analyses. Enzyme-linked immunosorbent assay (ELISA) kits were used to measure serum and urine levels of IP-10, galectin-9 and SIGLEC-1 according to the instructions of the manufacturers [IP-10: Human CXCL10/IP-10 Quantikine ELISA Kit, catalog number DIP100/SIP100/PDIP100, R&D Systems, Inc., Minneapolis, MN, USA; galectin-9: Human Galectin-9 Quantikine ELISA Kit, catalog number DGAL90, R&D Systems, Inc., Minneapolis, MN, USA; SIGLEC-1: Human Sialoadhesin (SIGLEC-1) ELISA Kit, catalog number KTE60663, Abbkine, Inc., Wuhan, PR China]. Serum and urine levels of IP-10 and SIGLEC-1 were reported as pg/ml, and galectin-9 as ng/ml. Also, urine levels of IP-10, galectin-9 and SIGLEC-1 were standardized according to creatinine concentrations in the spot urine specimens, and were reported as pg/mgCr, ng/mgCr and pg/mgCr, respectively.

### Statistical Analyses

Parametric and nonparametric tests were used according to the distribution pattern of the data. Results were expressed as mean ± standard deviation (SD) when normally distributed or as median [interquartile range (IQR), 25%–75%] otherwise. Categorical variables were shown as frequency (%). Comparisons of continuous variables between two groups were made by using *t*-tests or the Mann–Whitney *U*. Paired *t*-test or Wilcoxon signed-rank test was used to compare biomarker levels at baseline and after the treatment. Differences in proportions of different patient groups were compared using the chi-squared test. Relationships were determined by Pearson correlation coefficient or Spearman’s *rho*. Receiver operating characteristic (ROC) curves were used and area under the curve (AUC) values were calculated to assess the performances of serum and urine levels of IP-10, galectin-9 and SIGLEC-1 in predicting overall and renal disease activity in patients with SLE. Statistical analyses were performed with SPSS for Windows (SPSS version 25.0, IBM Corp., Armonk, NY, USA) and graphics were generated using MedCalc for Windows (MedCalc version 19.0, MedCalc Software, Ostend, Belgium). All analyses were two sided and a p value of 0.05 or less was considered as statistically significant.

## Supplementary Data

**Table S1:** Baseline demographic, clinical, laboratory, and histopathological features of patients with active renal SLE.

Characteristics	Active renal SLE (n = 31)
Female sex, n (%)	23 (74.2)
Age (years), median (IQR)	33 (26–47)
Disease duration (months), median (IQR)	23 (0–90)
Duration of nephritis (months), median (IQR)	0 (0–78)
Histopathology	
Class I LN, n (%)	0
Class II LN, n (%)	3 (9.7)
Class III LN, n (%)	5 (16.1)
*Class III+V LN, n*	1
Class IV LN, n (%)	17 (54.8)
*Class IV+V LN, n*	2
Pure class V LN, n (%)	6 (19.4)
Activity score, mean ± SD	7.1±4.2
Chronicity score, median (IQR)	1 (0–1)
SLEDAI, median (IQR)	14 (10–16)
Clinical SLEDAI, median (IQR)	12 (8–12)
Renal SLEDAI, median (IQR)	8 (4–12)
Hematuria, n (%)	22 (70.9)
Proteinuria, n (%)	31 (100)
Pyuria, n (%)	13 (41.9)
Serum creatinine (mg/dL), median (IQR)	0.7 (0.6–1.1)
eGFR (mL/min/1.73 m ^2^), median (IQR)	117.2 (64.1–126.6)
Serum albumin (g/dL), mean ± SD	3±0.8
Proteinuria (g/g), median (IQR)	2.1 (1.31–4.79)
Serum C3 (mg/dL), median (IQR)	52 (38–84)
Serum C4 (mg/dL), median (IQR)	6 (4–14)
Anti-dsDNA positivity *, n (%)	17/27 (62.9%)

**Abbreviations:** dsDNA: double-stranded DNA, eGFR: estimated glomerular filtration rate, IQR: interquartile range, LN: lupus nephritis, SD: standard deviation, SLE: systemic lupus erythematosus, SLEDAI: Systemic Lupus Erythematosus Disease Activity Index.

* Results were available for 27 patients (87.1%) at the time of sampling.

**Table S2:** Serum and urine levels of IP-10, galectin-9, and SIGLEC-1 in patients with active SLE compared to inactive SLE and healthy controls.

Biomarkers	Active Renal SLE (n = 31)	Active extrarenal SLE (n = 32)	p ^a^	Active SLE (n = 63)	Inactive SLE (n = 30)	p ^b^	Healthy control (n = 32)	p ^c^
sIP-10 (pg/mL), median (IQR)	194.2 (110–481.2)	313.9 (181.1–423.7)	0.24	279.4 (147.5–430.4)	173.4 (142.3–247.9)	**0.046**	74.4 (58.8–103)	**<0.001**
sGalectin-9 (ng/mL), median (IQR)	11 (7.4–14.2)	12.4 (8–14.1)	0.49	11.7 (7.5–14.1)	8.7 (7.5–10)	**0.03**	5.6 (4.6–6.6)	**<0.001**
sSIGLEC-1 (pg/mL), median (IQR)	181.2 (164.9–208.5)	180 (151.8–277.5)	0.91	181.2 (157.9–213.9)	182.6 (169.9–203.1)	0.69	258.5 (179–602.1)	**0.001**
uIP-10 (pg/mL), median (IQR)	44.7 (15.9–110.9)	25.9 (15.1–70)	0.47	34.5 (15.9–73.9)	20.8 (9.9–53.4)	0.09	12.2 (1.8–25.7)	**<0.001**
uGalectin-9 (ng/mL), median (IQR)	6.4 (2.2–13.7)	12.3 (6.3–18.1)	**0.02**	8.8 (4.1–18.1)	11.5 (7–15.1)	0.28	10.6 (5.6–17.4)	0.29
uSIGLEC-1 (pg/mL), median (IQR)	302.9 (215.2–347.4)	326.9 (248.7–438.1)	0.09	321.1 (236.3–370.9)	297.7 (247.7–371)	0.83	290 (205.2–323.6)	**0.047**
uIP-10 (pg/mgCr), median (IQR)	83.2 (33.1–180.3)	63.8 (45.1–102)	0.32	73.5 (40.9–136.9)	26.1 (18.1–55.2)	**<0.001**	16.5 (5.1–32.5)	**<0.001**
uGalectin-9 (ng/mgCr), median (IQR)	10.9 (6–26.1)	19.9 (10.5–36.5)	**0.02**	15.5 (9.6–32.1)	11.4 (8.8–19.5)	0.18	13.6 (11.3–22.1)	0.76
uSIGLEC-1 (pg/mgCr), median (IQR)	531.6 (418.3–980.8)	745.2 (333.8–1227.8)	0.45	619.7 (389.4–1056.6)	393.2 (248.6–715.8)	**0.006**	425.7 (264.7–925.9)	**0.04**
Serum C3 (mg/dL), median (IQR)	52 (38–84)	91 (66.5–102.3)	**0.001**	73 (49–99)	106 (88–121)	**<0.001**	-	-
Serum C4 (mg/dL), median (IQR)	6 (4–14)	16 (8–19.8)	**0.004**	9 (5–18)	19 (11–25)	**0.002**	-	-
Anti-dsDNA positivity, n (%)	17/27 (62.9)	18/22 (81.8)	0.15	35/49 (71.4)	3/4 (75)	0.88	-	-

a comparing active renal with active extrarenal SLE,

b comparing active SLE with inactive SLE,

c comparing active SLE with healthy control.

**Abbreviations:** dsDNA: double-stranded DNA, IP-10: interferon-inducible protein 10, IQR: interquartile range, s: serum, SIGLEC-1: sialic acid binding immunoglobulin-like lectin-1, SLE: systemic lupus erythematosus, u: urine.

**Table S3:** Serum and urine levels of IP-10, galectin-9, and SIGLEC-1 in patients with active renal SLE compared to those with inactive renal SLE, those with active renal AAV, and healthy controls.

Biomarkers	Active Renal SLE (n = 31)	Inactive Renal SLE (n = 15)	p ^a^	Active Renal AAV (n = 17)	p ^b^	Healthy Control (n = 32)	p ^c^
sIP-10 (pg/mL), median (IQR)	194.2 (110–481.2)	166.5 (122.7–188.6)	0.36	86.9 (68.9–151)	**0.001**	74.4 (58.8–103)	**<0.001**
sGalectin-9 (ng/mL), median (IQR)	11 (7.4–14.2)	8 (7–9.2)	0.09	11.9 (10.4–13.5)	0.64	5.6 (4.6–6.6)	**<0.001**
sSIGLEC-1 (pg/mL), median (IQR)	181.2 (164.9–208.5)	185.2 (175.4–203.6)	0.39	195.2 (176.5–258.1)	0.07	258.5 (179–602.1)	**0.002**
uIP-10 (pg/mL), median (IQR)	44.7 (15.9–110.9)	23.9 (9.5–80.6)	0.26	17.5 (4.4–49.5)	**0.06**	12.2 (1.8–25.7)	**<0.001**
uGalectin-9 (ng/mL), median (IQR)	6.4 (2.2–13.7)	10.8 (2.8–18.6)	0.16	0.8 (0.4–6.7)	**0.005**	10.6 (5.6–17.4)	0.07
uSIGLEC-1 (pg/mL), median (IQR)	302.9 (215.2–347.4)	290.5 (216.5–371)	0.88	439.5 (371.4–490.6)	**<0.001**	290 (205.2–323.6)	0.27
uIP-10 (pg/mgCr), median (IQR)	83.2 (33.1–180.3)	26.3 (20.9–58.4)	**0.006**	36 (8.9–96.9)	**0.042**	16.5 (5.1–32.5)	**<0.001**
uGalectin-9 (ng/mgCr), median (IQR)	10.9 (6–26.1)	10.4 (4.1–23.9)	0.72	1.6 (0.8–14.9)	**0.009**	13.6 (11.3–22.1)	0.22
uSIGLEC-1 (pg/mgCr), median (IQR)	531.6 (418.3–980.8)	304.1 (210.5–728.4)	**0.05**	865.7 (685.1–1219.4)	**0.004**	425.7 (264.7–925.9)	0.09
Serum C3 (mg/dL), median (IQR)	52 (38–84)	103 (73.6–120)	**0.001**	-	-	-	-
Serum C4 (mg/dL), median (IQR)	6 (4–14)	18 (7–24.5)	**0.006**	-	-	-	-
Anti-dsDNA positivity, n (%)	17/27 (62.9)	1/2 (50)	0.72	-	-	-	-

a comparing active renal SLE with inactive renal SLE,

b comparing active renal SLE with active renal AAV,

c comparing active renal SLE with healthy control.

**Abbreviations:** AAV: ANCA-associated vasculitis, IP-10: interferon-inducible protein 10, IQR: interquartile range, s: serum, SIGLEC-1: sialic acid binding immunoglobulin-like lectin-1, SLE: systemic lupus erythematosus, u: urine.

**Table S4:** Serum and urine levels of IP-10, galectin-9, and SIGLEC-1 in patients with SLE with regard to immunosuppressive use at baseline.

Biomarkers	Patients treated with immunosuppressives (n = 76)	Patients not treated with immunosuppressives (n = 17)	p
sIP-10 (pg/mL), median (IQR)	191.4 (148.9–378.1)	279.4 (141.1–473.9)	0.66
sGalectin-9 (ng/mL), median (IQR)	10.5 (7.6–13.7)	8.7 (6.3–11.4)	0.16
sSIGLEC-1 (pg/mL), median (IQR)	182.6 (163.6–210.3)	171.5 (162.9–203.8)	0.39
uIP-10 (pg/mL), median (IQR)	24.6 (13.9–66.4)	30.8 (16.9–167)	0.26
uGalectin-9 (ng/mL), median (IQR)	10.6 (4.5–18.1)	9.9 (3.9–15.9)	0.74
uSIGLEC-1 (pg/mL), median (IQR)	322.9 (242.2–371)	275.1 (223.7–370.9)	0.45
uIP-10 (pg/mgCr), median (IQR)	53.6 (26–97.5)	60 (24.6–163.1)	0.51
uGalectin-9 (ng/mgCr), median (IQR)	12.6 (9.3–31.2)	14.9 (9.5–19.4)	0.59
uSIGLEC-1 (pg/mgCr), median (IQR)	552.2 (354.2–982.6)	394.4 (245.9–923.8)	0.26

**Abbreviations:** IP-10: interferon-inducible protein 10, IQR: interquartile range, s: serum, SIGLEC-1: sialic acid binding immunoglobulin-like lectin-1, SLE: systemic lupus erythematosus, u: urine.

**Table S5:** Correlation of biomarkers with various clinical and laboratory features of patients with SLE (n = 93).

Features	sIP-10	uIP-10 (pg/mL)	uIP-10 (pg/mgCr)	sGalectin-9	uGalectin-9 (ng/mL)	uGalectin-9 (ng/mgCr)	sSIGLEC-1	uSIGLEC-1 (pg/mL)	uSIGLEC-1 (pg/mgCr)
Age	r = −0.11, p = 0.32	r = −0.16, p = 0.14	**r = −0.23, p = 0.03**	r = 0.13, p = 0.21	r = −0.07, p = 0.49	r = −0.09, p = 0.39	r = −0.06, p = 0.59	r = 0.06, p = 0.58	r = 0.03, p = 0.75
SLEDAI	r = 0.19, p = 0.07	**r = 0.26, p = 0.04**	**r = 0.48, p < 0.001**	r = 0.17, p = 0.11	r = −0.12, p = 0.24	r = 0.11, p = 0.31	r = −0.02, p = 0.87	r = 0.003, p = 0.98	**r = 0.27, p = 0.009**
Clinical SLEDAI	**r = 0.22, p = 0.036**	**r = 0.23, p = 0.03**	**r = 0.47, p < 0.001**	r = 0.19, p = 0.07	r = −0.09, p = 0.41	r = 0.11, p = 0.29	r = −0.03, p = 0.77	r = −0.05, p = 0.66	**r = 0.22, p = 0.04**
Renal SLEDAI	r = −0.04, p = 0.72	r = 0.16, p = 0.14	**r = 0.29, p = 0.006**	r = 0.03, p = 0.80	**r = −0.24, p = 0.02**	r = −0.13, p = 0.20	r = −0.01, p = 0.91	r = −0.12, p = 0.24	r = 0.10, p = 0.33
eGFR	r = 0.09, p = 0.40	r = 0.06, p = 0.57	r = 0.07, p = 0.50	r = −0.03, p = 0.79	**r = 0.21, p = 0.04**	r = 0.16, p = 0.12	r = 0.01, p = 0.96	r = −0.07, p = 0.49	r = −0.15, p = 0.15
Proteinuria	r = 0.07, p = 0.66	r = 0.22, p = 0.15	**r = 0.37, p = 0.01**	r = 0.19, p = 0.20	r = −0.18, p = 0.23	r = −0.03, p = 0.85	r = 0.03, p = 0.86	r = 0.11, p = 0.49	r = 0.26, p = 0.09
Serum albumin	r = −0.21, p = 0.22	r = −0.33, p = 0.06	**r = −0.46, p = 0.006**	r = −0.03, p = 0.89	r = 0.07, p = 0.69	r = −0.03, p = 0.87	r = −0.13, p = 0.46	r = −0.09, p = 0.57	r = −0.18, p = 0.31
C3	r = −0.16, p = 0.16	**r = −0.26, p = 0.02**	**r = −0.46, p < 0.001**	r = −0.05, p = 0.67	r = 0.17, p = 0.13	r = −0.01, p = 0.92	r = −0.04, p = 0.69	r = 0.09, p = 0.39	r = −0.16, p = 0.15
C4	r = −0.12, p = 0.28	**r = −0.22, p = 0.04**	**r = −0.38, p < 0.001**	r = −0.12, p = 0.27	r = 0.12, p = 0.29	r = −0.04, p = 0.69	r = −0.08, p = 0.49	r = −0.001, p = 0.99	r = −0.19, p = 0.07
Anti-dsDNA positivity	r = 0.02, p = 0.88	r = −0.02, p = 0.92	r = 0.03, p = 0.85	r = 0.13, p = 0.34	r = 0.02, p = 0.87	r = 0.06, p = 0.65	r = 0.02, p = 0.89	r = 0.25, p = 0.07	r = 0.20, p = 0.15

**Abbreviations:** dsDNA: double-stranded DNA, eGFR: estimated glomerular filtration rate, IP-10: interferon-inducible protein 10, s: serum, SIGLEC-1: sialic acid binding immunoglobulin-like lectin-1, SLE: systemic lupus erythematosus, SLEDAI: Systemic Lupus Erythematosus Disease Activity Index, u: urine.

**Table S6:** Serum and urine levels of IP-10, galectin-9, and SIGLEC-1 at baseline and after treatment in patients with active SLE (n = 41).

Biomarkers	Baseline	After treatment	p
sIP-10 (pg/mL), median (IQR)	279.4 (162.9–398.7)	181.7 (127.3–310.1)	0.06
sGalectin-9 (ng/mL), median (IQR)	11.5 (7.5–14.1)	6.1 (4.1–7.9)	**<0.001**
sSIGLEC-1 (pg/mL), median (IQR)	179.7 (155.9–213.9)	168.3 (148–206.5)	0.44
uIP-10 (pg/mL), median (IQR)	34.5 (15–71.4)	55 (46.8–61.2)	0.13
uGalectin-9 (ng/mL), median (IQR)	8.9 (3.4–18.1)	6.5 (2.7–8.1)	**0.005**
uSIGLEC-1 (pg/mL), median (IQR)	270.1 (224.4–366.9)	244.6 (206.4–273.5)	**0.02**
uIP-10 (pg/mgCr), median (IQR)	73.5 (29.3–117.2)	121.9 (81.6–183.6)	**0.007**
uGalectin-9 (ng/mgCr), median (IQR)	11.8 (8.6–31.5)	10.9 (6–14.2)	0.13
uSIGLEC-1 (pg/mgCr), median (IQR)	572.7 (371.1–990.2)	511.8 (360.1–825.3)	0.44
Serum C3 (mg/dL), median (IQR)	82 (51.5–103)	101 (84–110)	**0.04**
Serum C4 (mg/dL), median (IQR)	10 (5–20)	18 (12–22)	**0.004**
Anti-dsDNA positivity, n (%)	19/28 (67.9)	2/16 (12.5)	**0.002** *

**Abbreviations:** dsDNA: double-stranded DNA, IP-10: interferon-inducible protein 10, IQR: interquartile range, s: serum, SIGLEC-1: sialic acid binding immunoglobulin-like lectin-1, SLE: systemic lupus erythematosus, u: urine.

* Comparison was based on 13 patients who had anti-dsDNA measurements both at baseline and after treatment.

Figure S1 Serum **(a–c)** and urine **(d–f)** levels of all biomarkers in the active renal SLE, active extrarenal SLE, and active renal AAV groups. Detailed statistics are provided in the text and supplemental tables (AAV: ANCA-associated vasculitis, IP-10: interferon-inducible protein 10, SIGLEC-1: sialic acid binding immunoglobulin-like lectin-1, SLE: systemic lupus erythematosus).

Figure S2 ROC analyses of serum **(a)** and urine **(b)** IP-10, galectin-9 and SIGLEC-1 in discriminating disease activity in patients with SLE as compared to serum C3 and C4 (IP-10: interferon-inducible protein 10, ROC: receiver operating characteristics, s: serum, SIGLEC-1: sialic acid binding immunoglobulin-like lectin-1, SLE: systemic lupus erythematosus, u: urine).

Figure S3 ROC analyses of serum **(a)** and urine **(b)** IP-10, galectin-9 and SIGLEC-1 in discriminating disease activity in patients with SLE as compared to presence of anti-dsDNA antibodies. This comparison was performed based on the results of 53 patients (57%) with SLE whose anti-dsDNA results were available (dsDNA: double-stranded DNA, IP-10: interferon-inducible protein 10, ROC: receiver operating characteristics, s: serum, SIGLEC-1: sialic acid binding immunoglobulin-like lectin-1, SLE: systemic lupus erythematosus, u: urine).

Supplementary References1

GladmanDD
UrowitzMB
GoldsmithCH
FortinP
GinzlerE


The reliability of the Systemic Lupus International Collaborating Clinics/American College of Rheumatology Damage Index in patients with systemic lupus erythematosus
Arthritis and Rheumatology
1997
40
809
13
10.1002/art.1780400506
91535402

LeveyAS
StevensLA
SchmidCH
ZhangYL
CastroAF3rd


A new equation to estimate glomerular filtration rate
Annals of Internal Medicine
2009
150
604
12
10.7326/0003-4819-150-9-200905050-00006
19414839
PMC27635643

WeeningJJ
D’AgatiVD
SchwartzMM
SeshanSV
AlpersCE


The classification of glomerulonephritis in systemic lupus erythematosus revisited
Journal of the American Society of Nephrology
2004
15
241
50
10.1097/01.asn.0000108969.21691.5d
14747370


## Figures and Tables

**Table 1:** Baseline demographic, clinical, and laboratory features of patients with SLE and AAV.

Characteristics	Active SLE (n = 63)	Inactive SLE (N = 30)	p^x^	Active renal AAV (n = 17)	p^y^
Female sex, n (%)	54 (85.7)	25 (83.3)	0.76	7 (41.2)	**<0.001**
Age (years), median (IQR)	33 (26–47)	39 (30.8–47.3)	0.77	60 (48–65.5)	**<0.001**
Disease duration (months), median (IQR)	56 (0.5–115)	86 (45.5–169)	**0.01**	0 (0–18.5)	**0.001**
Immunosuppressive agents^a^, n (%)					
Hydroxychloroquine	39 (61.9)	26 (86.7)	**0.02**	-	-
Azathioprine	12 (19)	5 (16.7)	0.78	3 (17.6)	0.89
Mycophenolate mofetil	19 (30.2)	11 (36.7)	0.53	1 (5.9)	**0.04**
Corticosteroids	50 (79.4)	21 (70)	0.32	15 (88.2)	0.41
Dose of corticosteroids (mg/day metilprednisolone), median (IQR)	20 (4–40)	4 (2–4)	**<0.001**	40 (40–60)	**<0.001**
Rituximab^b^	4 (6.3)	0	0.16	2 (11.8)	0.45
Belimumab^c^	2 (3.2)	0	0.32	-	-
Methotrexate	0	1 (3.3)	0.15	-	-
Calcineurin inhibitors	0	1 (3.3)	0.15	-	-
cSLEDAI, median (IQR)	8 (4–12)	0 (0-0)	**<0.001**	-	-
SLICC/ACR damage score, median (IQR)	0 (0–1)	0 (0–1)	0.77	-	-
BVAS, mean ± SD	-	-	-	16.5±4.9	-
Serum creatinine (mg/dL), median (IQR)	0.6 (0.5–0.8)	0.7 (0.6–0.8)	0.38	4.4 (1.6–6)	**<0.001**
eGFR (mL/min/1.73 m^2^), median (IQR)	119.6 (92.2–131.2)	110.9 (96.2–122.3)	0.44	13.9 (7.6–46.1)	**<0.001**
Serum C3 (mg/dL), median (IQR)	73 (49–99)	106 (88–121)	**<0.001**	-	-
Serum C4 (mg/dL), median (IQR)	9 (5–18)	19 (11–25)	**0.002**	-	-

a at the time of enrollment,

b in the last six months,

c in the last three months.

x comparing active SLE with inactive SLE,

y comparing active SLE with active renal AAV.

**Abbreviations:** AAV: ANCA-associated vasculitis, ACR: American College of Rheumatology, BVAS: Birmingham Vasculitis Activity Score, eGFR: estimated glomerular filtration rate, IQR: interquartile range, SLE: systemic lupus erythematosus, SD: standard deviation, cSLEDAI: Clinical Systemic Lupus Erythematosus Disease Activity Index, SLICC: Systemic Lupus International Collaborating Clinics.

**Table 2:** Serum and urine levels of IP-10, galectin-9 and SIGLEC-1 compared to C3, C4 and anti-dsDNA antibodies in patients with SLE (n = 93).

Biomarkers	Active SLE (n = 63)	Inactive SLE (n = 30)	p
sIP-10 (pg/mL), median (IQR)	279.4 (147.5–430.4)	173.4 (142.3–247.9)	**0.046**
sGalectin-9 (ng/mL), median (IQR)	11.7 (7.5–14.1)	8.7 (7.5–10)	**0.03**
sSIGLEC-1 (pg/mL), median (IQR)	181.2 (157.9–213.9)	182.6 (169.9–203.1)	0.69
uIP-10 (pg/mL), median (IQR)	34.5 (15.9–73.9)	20.8 (9.9–53.4)	0.09
uGalectin-9 (ng/mL), median (IQR)	8.8 (4.1–18.1)	11.5 (7–15.1)	0.28
uSIGLEC-1 (pg/mL), median (IQR)	321.1 (236.3–370.9)	297.7 (247.7–371)	0.83
uIP-10 (pg/mgCr), median (IQR)	73.5 (40.9–136.9)	26.1 (18.1–55.2)	**<0.001**
uGalectin-9 (ng/mgCr), median (IQR)	15.5 (9.6–32.1)	11.4 (8.8–19.5)	0.18
uSIGLEC-1 (pg/mgCr), median (IQR)	619.7 (389.4–1056.6)	393.2 (248.6–715.8)	**0.006**
Serum C3 (mg/dL), median (IQR)	73 (49–99)	106 (88–121)	**<0.001**
Serum C4 (mg/dL), median (IQR)	9 (5–18)	19 (11–25)	**0.002**
Anti-dsDNA positivity*, n (%)	35/49 (71.4)	3/4 (75)	0.88

**Abbreviations:** dsDNA: double-stranded DNA, IP-10: interferon-inducible protein 10, IQR: interquartile range, s: serum, SIGLEC-1: sialic acid binding immunoglobulin-like lectin-1, SLE: systemic lupus erythematosus, u: urine.

* Results were available for 53 patients with SLE at the time of sampling, 49 of whom had active disease.
