# Supplementary figures and images for: Serum and urine interferon-inducible protein 10, galectin-9, and SIGLEC-1 as biomarkers of disease activity in systemic lupus erythematosus
Source: Turk J Med Sci. 2024 Jan 20;54(2):391–400. doi: 10.55730/1300-0144.5804 (PMC11265893; doi:10.55730/1300-0144.5804)

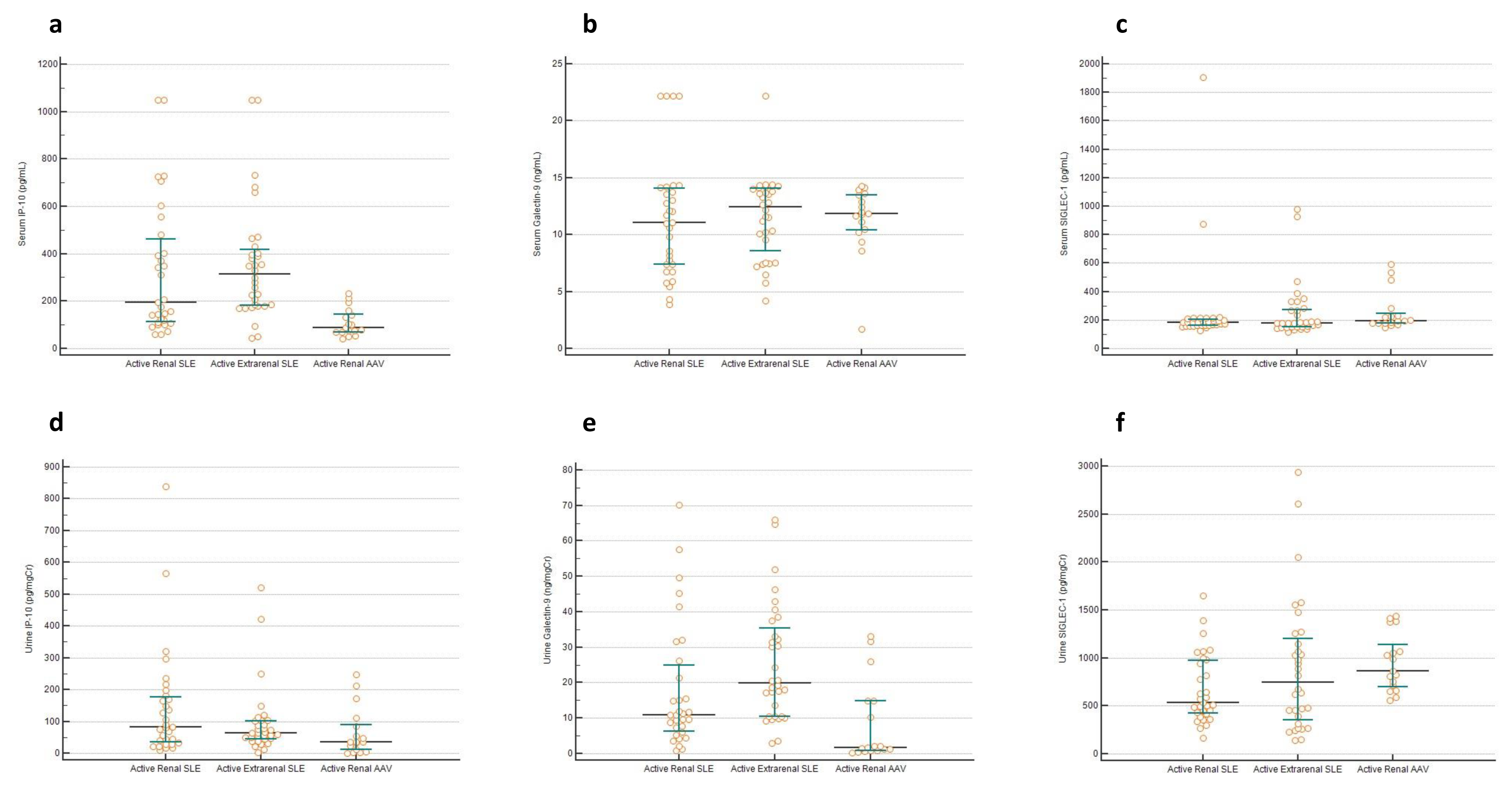

Supplement: Figure S1 — Serum (a–c) and urine (d–f) levels of all biomarkers in the active renal SLE, active extrarenal SLE, and active renal AAV groups. Detailed statistics are provided in the text and supplemental tables (AAV: ANCA-associated vasculitis, IP-10: interferon-inducible protein 10, SIGLEC-1: sialic acid binding immunoglobulin-like lectin-1, SLE: systemic lupus erythematosus). [file tjmed-54-02-391s1.tif]

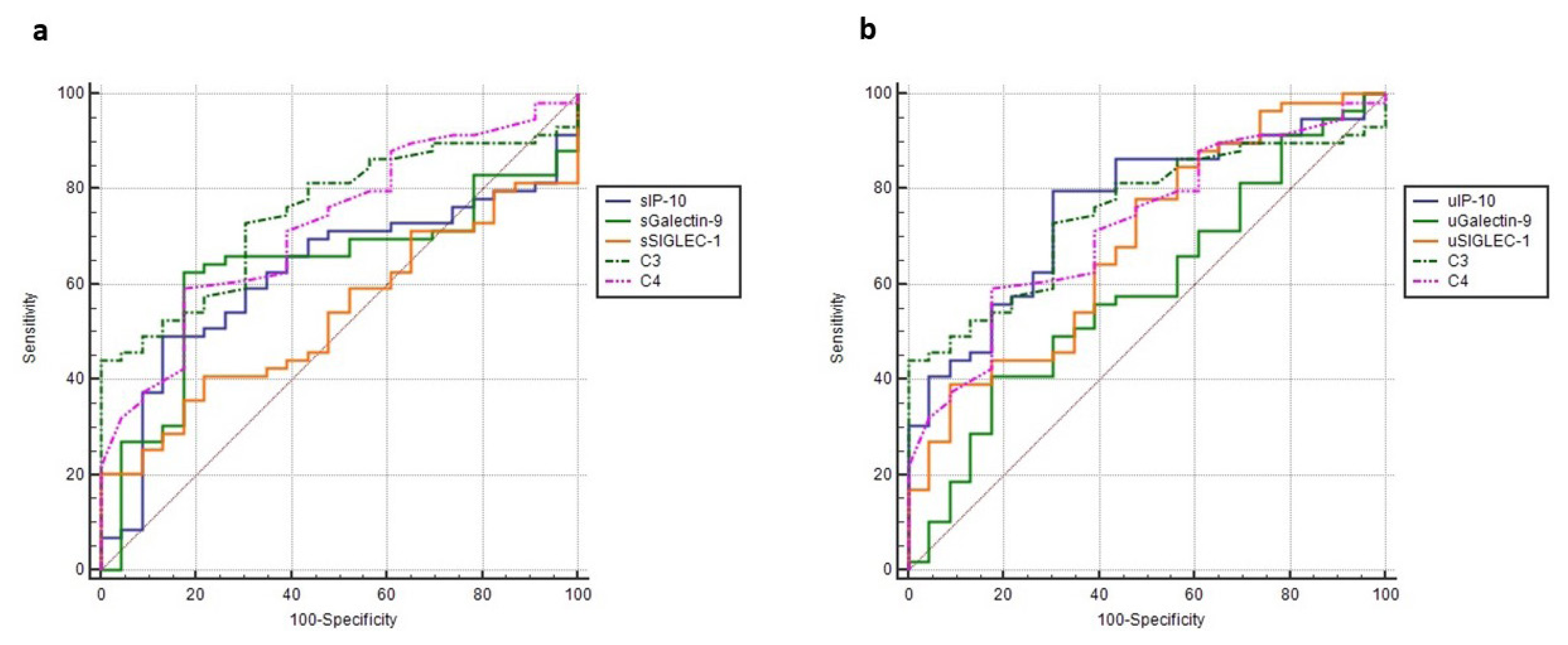

Supplement: Figure S2 — ROC analyses of serum (a) and urine (b) IP-10, galectin-9 and SIGLEC-1 in discriminating disease activity in patients with SLE as compared to serum C3 and C4 (IP-10: interferon-inducible protein 10, ROC: receiver operating characteristics, s: serum, SIGLEC-1: sialic acid binding immunoglobulin-like lectin-1, SLE: systemic lupus erythematosus, u: urine). [file tjmed-54-02-391s2.tif]

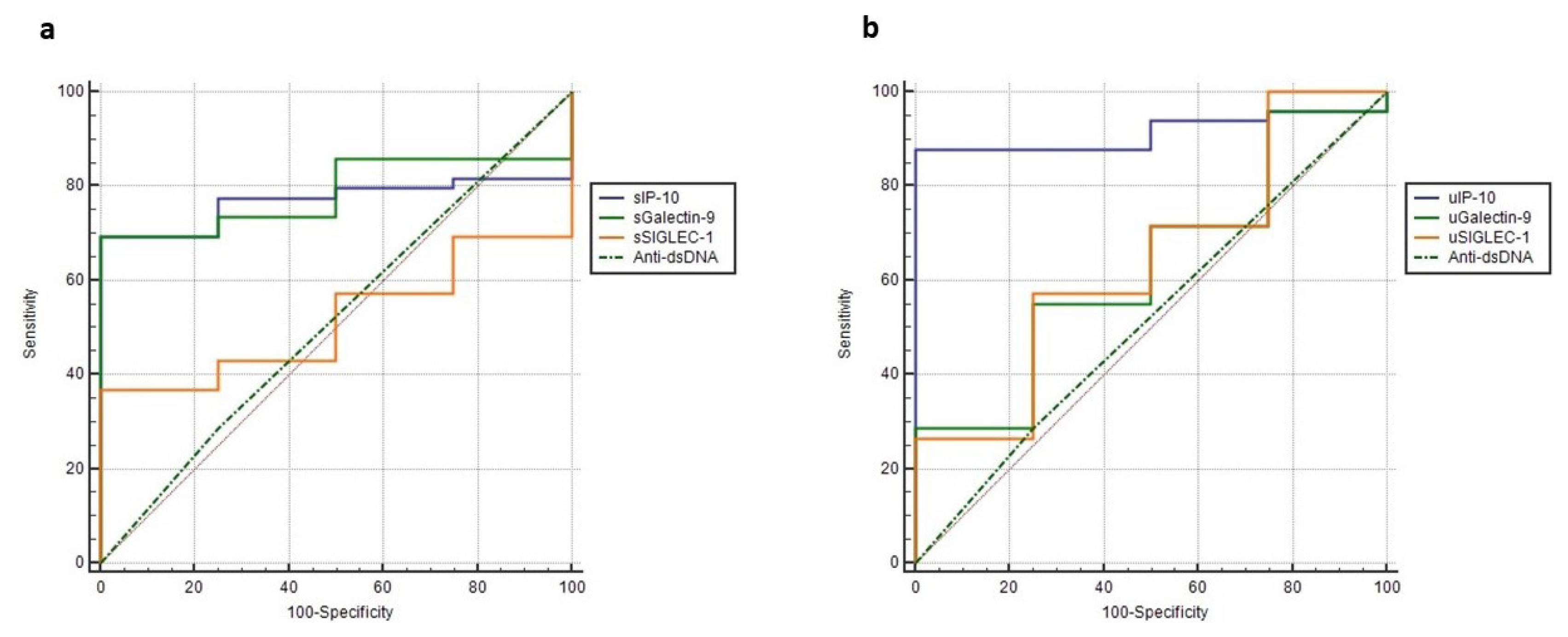

Supplement: Figure S3 — ROC analyses of serum (a) and urine (b) IP-10, galectin-9 and SIGLEC-1 in discriminating disease activity in patients with SLE as compared to presence of anti-dsDNA antibodies. This comparison was performed based on the results of 53 patients (57%) with SLE whose anti-dsDNA results were available (dsDNA: double-stranded DNA, IP-10: interferon-inducible protein 10, ROC: receiver operating characteristics, s: serum, SIGLEC-1: sialic acid binding immunoglobulin-like lectin-1, SLE: systemic lupus erythematosus, u: urine). [file tjmed-54-02-391s3.tif]
